# Complicated Herniæ

**Published:** 1895-04

**Authors:** S. E. Milliken

**Affiliations:** New York


### COMPLICATED HERNIAE.

Dr. S. E. Milliken, of New York, in a paper upon this always interesting subject, read before the Alumni Association, of the Northern Dispensary in November, and subsequently published in the *New York Medical Journal*, of March 16, 1895, described some of the complications frequently met with in the diagnosis and treatment of inguinal hernia, which, while not dangerous, were nevertheless important. These were, "Adhesions," "Undescended testis," and "Hydrocele of the cord."

ADHESION is most commonly omental, the intestine being rarely involved. It often escapes detection, owing to the absence of symptoms. It may be demonstrated, by first reducing the hernia by taxis, and then, by making traction on the cord, reproducing it. Radical operation is advised, the method of Bassini being preferred.

UNDESCENDED TESTIS does not occur so frequently. It is readily recognized. To relieve it the testis must be freed from adhesions, and anchored in the scrotum. The canal is reconstructed after the method of Bassini.

ENCYSTED HYDROCELE OF THE CORD is more often mistaken for hernia than any other condition. When small it can be reduced into the canal, and will reappear if traction is made on the cord. It can readily be differentiated from hernia, however, by the absence of pain, and the firmness of the swelling. Aspiration and the injection of carbolic acid is recommended.

CONCLUSIONS:

1—Besides becoming strangulated, hernia may be complicated by adhesions, undescended testis, and hydrocele of the cord.

2—Omentum often becomes adherent without causing any alarming symptoms, and is the greatest obstacle in the way of successful mechanical treatment.

3 The caecum may take on adhesions under an ill-fitting truss, and yet not become strangulated.

4—Undescended testis rarely exists alone, and is usually complicated by hernia.

5—Encysted hydrocele of the cord, while often mistaken for hernia, may be only a complication.

